# Impact of sediment mobilization on trace elements release in Galician Rías (NW Iberian Peninsula): insights into aquaculture

**DOI:** 10.1007/s10661-024-12950-2

**Published:** 2024-08-24

**Authors:** Belén Rubio, Ángel Enrique López-Pérez, Iván León

**Affiliations:** 1 Centro de Investigación Mariña, Universidade de Vigo, GEOMA, 36310 Vigo, Spain; 2 Facultad de Ciencias Básicas, Universidad del Atlántico, Grupo de Zona Costera, Barranquilla, Colombia

**Keywords:** Aquaculture, Trace element speciation, Aerobic oxidation, Metal toxicity, Ría de Pontevedra

## Abstract

In the latest years, the concentration levels of certain metals and metalloids in the sediments of the Galician Rías have shown an increasing trend (e.g., As, Zn, Cu, Pb, Hg). These areas are also characterized by their richness in nutrients and their great aquaculture or mariculture activity, with the presence of more than 3500 mussel rafts in the Rías Baixas. The inner areas of the Galician Rías are subjected to activities that resuspend the sediment such as high levels of maritime traffic and dredging or cleaning operations. It is likely that a transfer of these elements to the water column happens during the resuspension of sediments caused by natural events or anthropogenic activities. In this study, selected samples of surface sediments of the Ría de Pontevedra (NW Spain) were subjected to a procedure of aerobic oxidation to determine the concentration of some elements (Fe, Mn, Cu, Cr, Pb, Hg, and Zn) released from the sediment to the aqueous phase. The experiment was carried out within 5 days. Measurements of pH and total concentration were taken both in water and sediment samples. Furthermore, speciation of trace elements was carried out in the sediment samples. Trace element concentrations were lower in the sediments during aerobic oxidation, being released to the aqueous phase. From an environmental point of view, Cu was the only trace element released in quantities that may be toxic for the organisms in the area. This problem of sediment oxidation related to dredging activities or natural storm conditions should be considered in environmental impact studies and transferred to stakeholders.

## Introduction

Metal pollution has been reported in sediments and water column from the Galician Rias for the last decades (Beiras et al., 2003a, b; Prego and Cobelo-García, 2003; Cobelo-García and Prego, 2004; Prego et al., 2006; Álvarez-Iglesias et al., 2006, 2012; Bellas et al., 2008, 2011; Santos-Echeandía et al., 2008a, b; Álvarez-Iglesias & Rubio, 2009; Durán & Nieto, 2011; Rodríguez-Germade et al., 2014; Monaco et al., 2017; Guevara et al., 2021; Gardoki et al., 2023; Otero et al., 2024). Previous studies of metal speciation in the sediments of the Galician Rias have identified the geochemical fractions in which certain trace elements are found and have provided valuable information on the geochemical and environmental status of these sediments (Cela et al., 1992; Belzunce-Segarra et al., 1997, 2008; Prego and Cobelo-García, 2003; Álvarez-Iglesias et al., 2003; Villares et al., 2003; Rey et al., 2005; Ramírez-Pérez et al., 2017, 2020). In these sediments, a significant fraction of certain elements is concentrated mainly in the non-residual fraction, especially for the most harmful metals and metalloids such as Cu, Pb, As, and Hg (Álvarez-Iglesias & Rubio, 2008; León et al., 2004; Rubio et al., 2001). Although, in general, these elements are found in low proportions in the most labile fractions, in the inner areas of the rías, where the sediments are suboxic-anoxic, they are also concentrated in relatively high proportions in the oxidizable fractions of the sediment (sulfides and organic matter).

Aerobic oxidation of iron sulfides and organic matter, because of bioturbation or resuspension of the sediment, can lead to trace elements associated with these geochemical fractions being released into the water column (Huerta-Diaz et al., 1998; Otero et al., 2005). In general, anthropogenic activities have the greatest potential for such resuspension. There are usually many activities in the rias that contribute to resuspension. One of these is port activities, whose maritime traffic has increased considerably in recent decades (García et al., 2013; Navarro, 2000; Pérez-Cid et al., 2021). Others correspond to the cleaning and care activities of mussel cultivation in rafts, which also favor sediment resuspension, such as the mobilization of the large amount of bio-deposits, generally rich in trace elements, that they produce (Cabanas et al., 1979; Calvo de Anta et al., 1999; León et al., 2004; Otero et al., 2009; Rubio et al., 2011).

Furthermore, sediment dredging is a common practice in the ports of the Galician Rías. In the region of Galicia, dredging operations are regulated by the Law 6/2017, of December 12, on ports of Galicia. Studying the behavior and risk of trace elements during dredging activities is essential, as they have the potential to greatly influence the mobility of these elements within sediments, leading to their release into the water column (Ferrans et al., 2021; Torres et al., 2009). Galicia is a region where this type of dredging information is not stored in scientific publications or public archives. However, these dredging activities in the Ría of Pontevedra are frequently reported in the press. For instance, between 1990 and 2010, the port of Marín in the Ría of Pontevedra conducted eight dredging operations, displacing a total of 139,000 cubic meters of seabed material (https://www.farodevigo.es/pontevedra/2010/11/26/dragados-puerto-supusieron-15-anos-17798645.html). There are few scientific works that address the evaluation of the environmental impact of dredging in the Galician Rías. Rodríguez-Romero et al. (2016) evaluated the effect of dredging in Ría of Arousa, showing that the sediments in the study were not highly contaminated.

Understanding desorption/adsorption processes is also a fundamental requirement for understanding the geochemical characteristics of the environment, and the release mechanisms and the transport, mobility, and bioavailability patterns of these trace elements and metalloids.

For a proper environmental management decision-making, it is very important during the assessment process to know the mobility and possible adsorption of these elements, as these processes in the sediment are complex due to rapidly changing conditions. To understand these mechanisms, laboratory studies are conducted, subjecting both sediment and solution to various physicochemical conditions. Typically, the results are measured in the aqueous phase (Caille et al., 2003; Gambrell et al., 1991; Otero et al., 2005), but very little work has focused on the study of the variation in the distribution of the geochemical fractions of the sediment (e.g., Álvarez-Iglesias et al., 2003; Belzunce-Segarra et al., 2008; Madadi et al., 2023; Otero et al., 2005; Ramírez-Pérez et al., 2020), and particularly the studies focused in this distribution after oxidation are very scarce.

This research is focused on offering an integrated approach that links the transfer processes of trace elements between sediment and the water column. Additionally, it aims to identify geochemical oxidizable fractions through aerobic oxidation. The assessment of trace elements released shall be done by speciation of the elements in the sediment and by measuring the concentration of the elements in the aqueous phase. In addition, the potential hazard of the levels of trace elements released into the aqueous phase shall be assessed.

## Study area

**Fig. 1:**
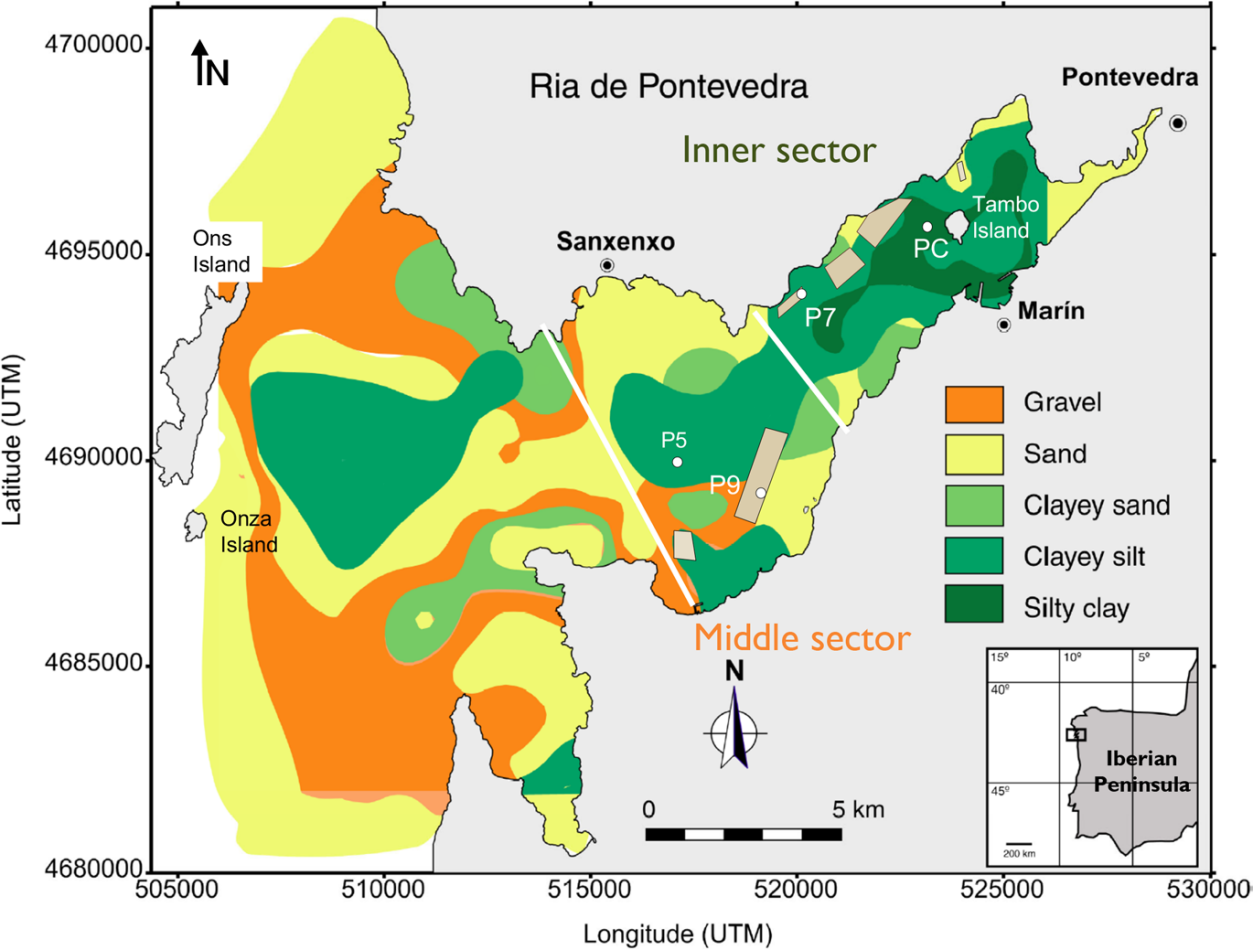
Map of location and granulometric distribution of the Ría de Pontevedra, showing the positions of samples P5 and P9 (middle sector) and P7 and PC (inner sector). The brown rectangles represent the areas with mussel rafts. Modified from Vilas et al., (1996)

The ria de Pontevedra, located at the NW of the Iberian Peninsula, is about 30 km long and has a maximal width of 12 km at the mouth (Fig. 1). Geomorphologically, the ría can be divided into three sectors: the inner, the middle, and the outer, where it reaches a maximum depth of 60 m (Hernández-Otero et al., 2014). The ría is characterized by the presence of three islands: Tambo Island in the inner sector, and Ons and Onza Islands in the outer sector.

The region has a humid climate, with average summer temperatures around 19 °C and winter temperatures around 10 °C. Annual rainfall averages approximately 1000 mm (Rubio et al., 2001). The storm regime in the study area is controlled by the position of the Azores anticyclone. In summer, anticyclonic conditions prevail along the Galician coast, leading to northerly winds and little precipitation. During the winter months, the Azores anticyclone moves to lower latitudes, resulting in low-pressure conditions and the formation of Atlantic fronts with southwest winds, increasing storms and precipitation (Vilas et al., 1996).

The Ría de Pontevedra exhibits a mesotidal regime, featuring an average tidal range of 2.2 m and a semidiurnal tidal cycle (Rubio et al., 2001; Vilas et al., 2019). In the Galician Rias, tidal effects on the transport of sediments and post-sedimentary processes are recognizable in the inner most part; meanwhile in the external and central sectors, the tidal has a limited influence (Rey et al., 2005; Vilas et al., 2005, 2019). Furthermore, currents in the inner sector are influenced by factors such as river discharge, primarily from the Lérez River and wind (Prego et al., 2001). Additionally, the inner sector is more influenced by anthropogenic inputs and has a higher content of mud and organic matter compared to the other sectors of the estuary (Rubio et al., 2001).

The Ría de Pontevedra is also under the influence of an oceanic coastal circulation, such as upwelling and downwelling processes. The hydrography of the Ría is affected by upwelling conditions through the East North Atlantic Central Water (ENACW) during the summer when the dominant winds have a northerly component. In winter, the prevailing winds are from the southwest, influencing the Iberian Poleward Current (IPC) and the downwelling processes (Prego et al., 2001; Rubio et al., 2001; Vilas et al., 2019). Furthermore, there is an intense swell during winter conditions, reaching significant maximum wave heights (Hs_max_) of around 8 m in the Galician Rías (Vilas et al., 2019). Numerical simulations conducted by Rey et al. (2005) indicate that sediment remobilization occurs with wave heights of 2.5 m and periods of 14 s, resulting in seabed sediment oxygenation.

In general, water circulation is marked by a prevalence of freshwater along the northern margin and marine water dominance along the southern margin (Rubio et al., 2001). Moreover, the sedimentation rates on the Ría de Pontevedra are about 1 mm year^−1^ (Rubio et al., 2001). Its sedimentary dynamics share similarities with wave-dominated estuaries, albeit with lower input of freshwater from the continent, and increased primary productivity attributable to the seasonal upwelling described (Rey et al., 2005). Waves play an important role in the distribution of sediments. In general, wave energy is lower in the axial region owing to the increased water depth, and in protected areas of the inner sector. These low-energy areas are characterized by the accumulation of fine-grained and organic-rich material. In contrast, in high-energy areas, such as the margins of the Ría and the outer sector, coarse-grained, carbonate-rich sediments are accumulated (Rey et al., 2005).

## Material and methods

**Fig. 2:**
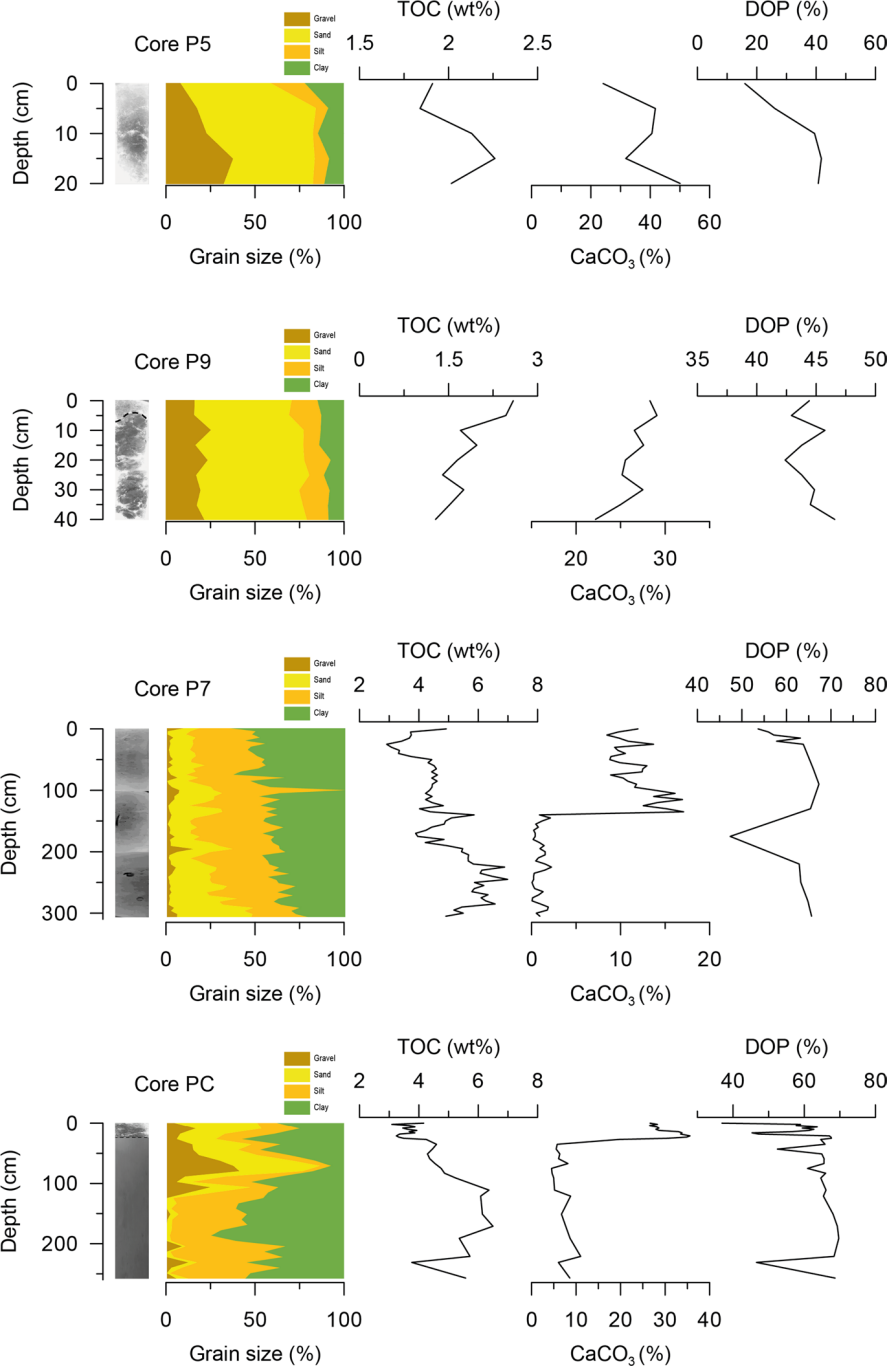
Plot of the grain-size distribution for the cores P5, P9, P7, and PC along with the percentage of organic carbon, carbonates, and DOP

**Fig. 3:**
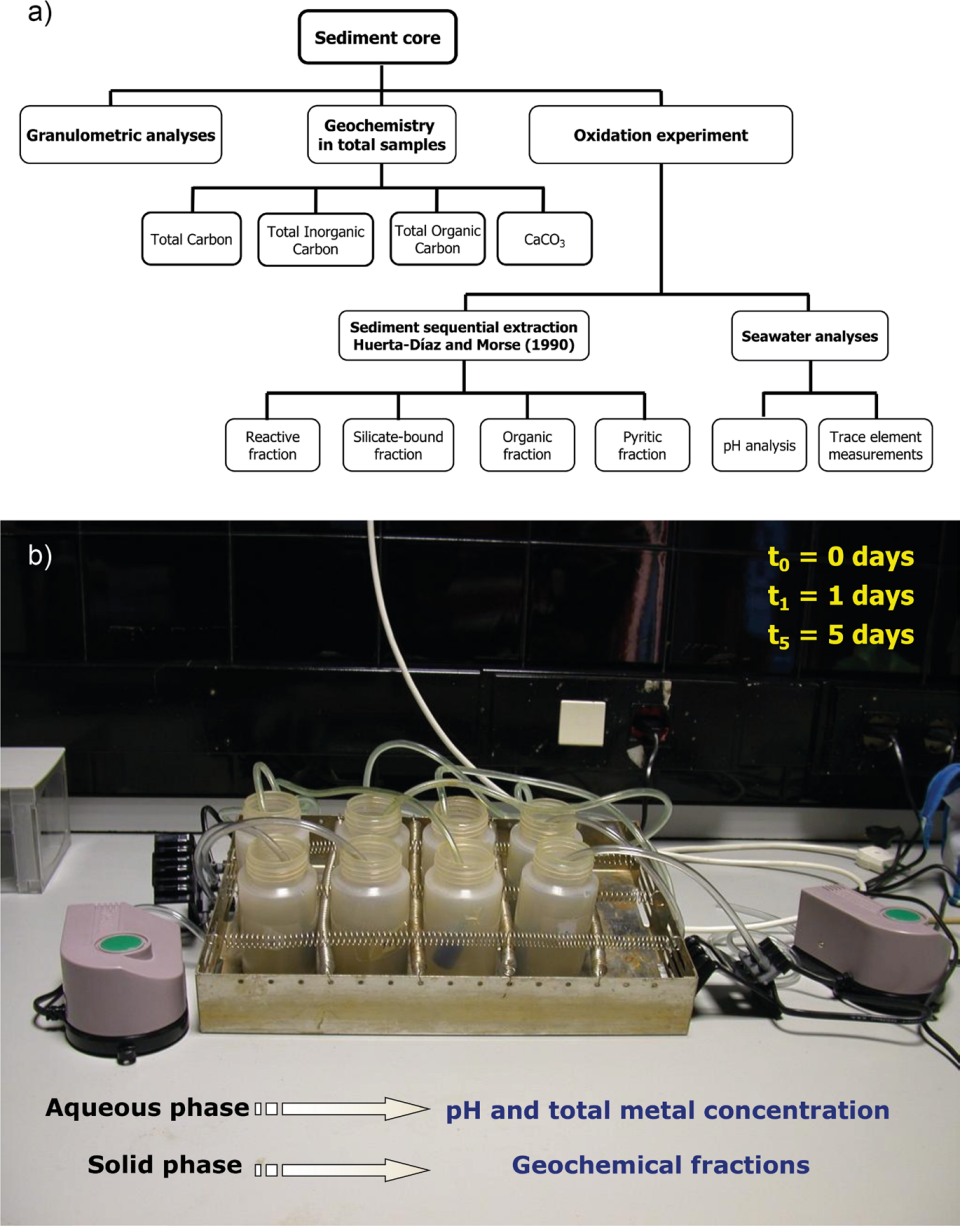
**a** Flow chart with the methodology used in this work. **b** Photograph of the experimental setup where the samples were subjected to oxidation using an aquarium pump during 5 days

Surface sediment samples (0–5 cm) from gravity corers collected in the inner area (cores P7 and PC) and in the middle area (cores P5 and P9) from the Ría de Pontevedra (NW Spain) were selected based on their grain-size and physico-chemical characteristics (Figs. 1 and 2). The methodological strategy is shown in Fig. 3a, b.

Sediment cores were obtained using a gravity corer, approximately 4.5 m in length and 12 cm in diameter, with an inner PVC tube that collects the sediment. They were collected from depths between 20 and 40 m, with lengths varying from 0.25 to 3.06 m. In the laboratory, the cores were stored in a refrigerated chamber at a temperature below 4 °C until analysis.

In terms of grain-size analyses, the samples were rinsed with water over a sieve with a 0.063 mm diameter to segregate the larger fractions from the finer ones. The particle size analysis of the larger fractions was conducted via dry sieving utilizing a set of sieves, each one phi. The total content of the fine fraction was determined using the Robinson pipette method. Finally, the analysis of fine grain size (< 0.063 mm) was performed using a Sedigraph 5100. The coarse fraction was considered to be anything larger than 2 mm, the sand fraction between 0.063 and 2 mm, and the fine fraction smaller than < 0.063 mm.

The contents of total carbon (TC) and total inorganic carbon (TIC) were determined using a Carlo-Erba™ elemental analyzer. The total organic carbon (TOC) was calculated by the difference between the TC and TIC contents.

The percentage of CaCO_3_ was estimated using the equation TIC × 100/12, where 100/12 is the molecular weight ratio of CaCO_3_ to carbon.

For the oxidation experiment, duplicates of the surface samples of the cores were placed in plastic bottles containing 100 ml of filtered ria seawater, which had previously undergone an ageing process for 1 week. The seawater was collected from the Ría de Pontevedra at a depth of 25 m. The sediments were kept in suspension by mounting the flasks in a Variomag Electronicrührer single-stage shaker. During the experiment, air with oxygen was pumped into the system with an aquarium pump (Fig. 3b).

The experiment was conducted over a time interval of 5 days. During this time, three measurements were made. The first determination was made at time zero (t0, day 0), the second at 24 h (t1, day 1), and the last at 120 h (t5, day 5).

In the seawater, pH and total trace element concentration were measured directly using a *Metrohm* 826 pH mobile electrode. The pH in the aqueous phase was measured using a glass electrode calibrated with analytical-grade buffer solutions. The remobilization experiment was conducted in the dark, at ambient temperature and atmospheric pressure. The bottles were sealed with paraffin wax, with small holes punctured to facilitate gas exchange while minimizing evaporation. T0 represents the measurement of trace elements in seawater prior to the experiment. On both day 1 and day 5, samples were withdrawn and centrifuged in a Tornax at 4000 rpm for a 30-min interval. The samples were filtered through 0.45 µm Millipore filters. The filtered samples were stored in plastic bottles, and the total concentrations of dissolved trace metals were immediately analyzed. The residue remaining from the filtration was washed twice with deionized water to remove present salts. Sequential extraction was performed on this residue to identify the geochemical fractions of trace metals (Huerta-Diaz & Morse, 1990). Sediment oxidation was calculated through the variation in metal concentrations in the geochemical fractions determined by the method used.

In the sediment, the sequential extraction of trace elements proposed by Huerta-Diaz & Morse, 1990) was carried out, in which four geochemical fractions were obtained: reactive (F_react_), organic (F_mo_), pyritic (F_pyr_), and silicate-bound (F_sil_). The oxidation of the sediments was calculated through the variation of the trace element concentrations in the geochemical fractions determined by the method used. The extractants used are shown in Table 1. Due to the high organic matter content in the analyzed sediments, concentrated H_2_SO_4_ was used as suggested by these authors. The analyses were conducted on 1 g of dry sediment sample in a fraction smaller than 0.063 mm. The procedure is as follows:

1. Reactive fraction (F_react_): Metals were extracted with 20 ml of 1N HCl after 16 h of continuous agitation at room temperature.
2. Fraction associated with aluminosilicates (F_silic_): To the previous residue, 30 ml of 30 M HF was added for 16 h of continuous agitation at room temperature.
3. Fraction associated with organic matter (F_mo_): The residue from Fsilic was contacted with 10 ml of concentrated H_2_SO_4_ for 2 h of continuous agitation at room temperature.
4. Pyritic fraction or sulfide-associated fraction (F_pyr_): Finally, metals associated with sulfides were extracted with 10 ml of concentrated HNO_3_ for 2 h of continuous agitation at room temperature.

**Table 1:** Extractants used for the sequential extraction of metals according to the method described by Huerta-Díaz and Morse (1990)

Fraction	Extractant	Sediment components
Reactive Fe	20 ml HCl	Exchangeable ions, amorphous Fe oxides and hydroxides, carbonates, and acid-volatile sulfides (AVS)
Silicate-bound Fe	30% v/v HF	Lithogenic minerals
Organic matter-bound Fe	H_2_SO_4_-concentrated	Organic matter
Pyritic Fe	HNO_3_	Pyrite

At the end of each extraction, the samples were centrifuged at 4000 rpm (Tornax model) for 30 min. The supernatant was collected with a pipette and put into bottles for heavy trace metal analysis. The residue was washed with 8 ml of Milli-Q water and centrifuged again for 30 min at 4000 rpm. This second supernatant was discarded.

For each set of 7 samples, one sample was introduced in triplicate to verify the accuracy and reproducibility of the method. In all cases, the accuracy was within ± 2% at a confidence level of 95%. The results are expressed in milligrams per kilogram of dry sediment (mg kg^−1^), except for Fe (mg g^−1^) and Hg (µg kg^−1^). In seawater, they are expressed in µg l^−1^.

$$\text{The degree of pyritization }(\text{DOP})\text{ was determined using the formula DOP}=\frac{Fpyr}{Fpyr+ Freactive}\times 100$$

Trace elements in sediment extracts and seawater were analyzed using inductively coupled plasma atomic emission spectroscopy (ICP-AES; model PerkinElmer Optima 4300 DV). Mercury concentration was analyzed by cold vapor atomic absorption spectrometry (CV-AAS) on a PerkinElmer FIMS 400. The detection limits of trace elements in sediment fractions and seawater are displayed in Table 2.

**Table 2:** Detection limits (mg kg^−1^) of trace elements: (a) in fractions defined by the method of Huerta-Díaz and Morse (1990); (b) in seawater

(a)					
Trace elements	Detection limits (mg l^−1^)* in sediment fractions				
	Reactive		Residual	Organic	Pyritic
Fe	0.019		0.261	0.067	0.031
Mn	0.001		0.001	0.013	0.001
Cu	0.003		0.002	0.002	0.004
Cr	0.007		0.012	0.008	0.005
Ni	0.016		0.018	0.090	0.002
Pb	0.010		0.012	0.001	0.001
Zn	0.011		0.008	0.030	0.003
As	0.040		0.290	1.050	0.050
Hg	0.020		0.019	0.200	0.057
* except Hg (µg l^−1^)					
b)					
Trace elements		Detection limits in seawater			
		(µg l^−1^)			
Fe		0.1			
Mn		0.1			
Cu		0.4			
Cr		0.2			
Pb		0.1			
Zn		0.001–0.005 mg l^−1^			
Hg		< 0.01–0.02			

## Results

### Grain size and chemical characterization

On average, inner area samples (cores P7 and PC) are muddy and richer in organic matter than the middle area ones (cores P5 and P9) with a high sand percentage and a lower organic matter content (Fig. 2). Cores P7 and PC display the highest percentages of the fine fraction (77.40% ± 9.70% and 75.24% ± 25.29%, respectively) and TOC (4.89% ± 0.98% and 4.45% ± 1.00%), and the lowest content of carbonates (5.84% ± 5.79% and 17.48% ± 12.04%, respectively) (Fig. 2).

In contrast, cores P5 and P9 show high percentages of sand (54.48 ± 8.49 and 56.58 ± 3.79, respectively) and CaCO_3_ (37.63 ± 9.98 and 26.32 ± 2.11, respectively) and the lowest content of TOC (2.03 ± 0.17 and 1.82 ± 0.45, respectively) (Fig. 2).

Considering the degree of pyritization (DOP) classification from León et al., (2004), the cores from the inner area of the Ría are anoxic, whereas the middle area ones are oxic-suboxic (Fig. 2). Cores P7 and PC exhibit the highest values of DOP (61.21% ± 5.61% and 61.49% ± 8.20%, respectively), in comparison to P5 and P9 (32.79% ± 11.36% and 44.33% ± 1.31%, respectively) (Fig. 2). This classification is derived from sedimentary DOP values obtained through HCl extraction. According to this classification, the sedimentary environment is considered oxic when DOP values are < 42%, dioxic or suboxic when they fall between 42 and 55%, anoxic between 55 and 75%, and euxinic when DOP exceeds 75% (León et al., 2004).

### Aqueous phase: pH and trace elements variations

**Fig. 4:**
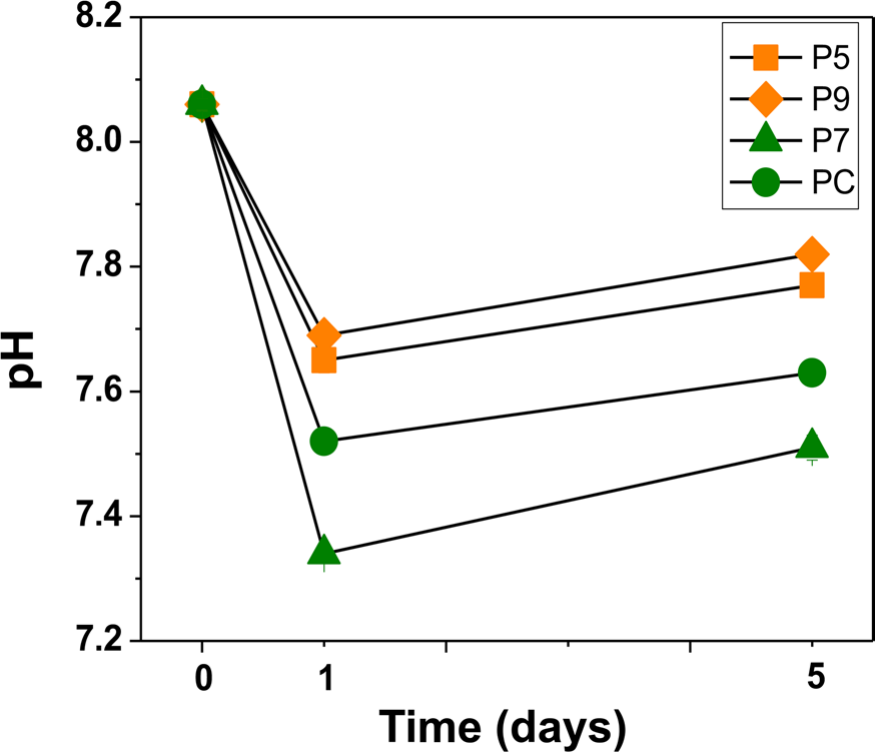
Temporal variation of pH in the aqueous phase during the aerobic resuspension of sediments from the Ría de Pontevedra

The temporal variation of pH in seawater shows a net decrease from the beginning to the end of the experiment (Fig. 4). During the initial stage of the experiment (from the beginning to day 1), there was a considerable decrease in pH in all samples, with the strongest decrease being reached in sample P7, the sample collected below the mussel rafts. On day 5, a slight increase in pH was observed in all samples. However, the values were still lower than the initial pH values of the experiment. In general, this decrease was more pronounced for samples from the inner sector (P7 and PC) than for those from the middle sector (P5 and P9) (Fig. 4).

**Fig. 5:**
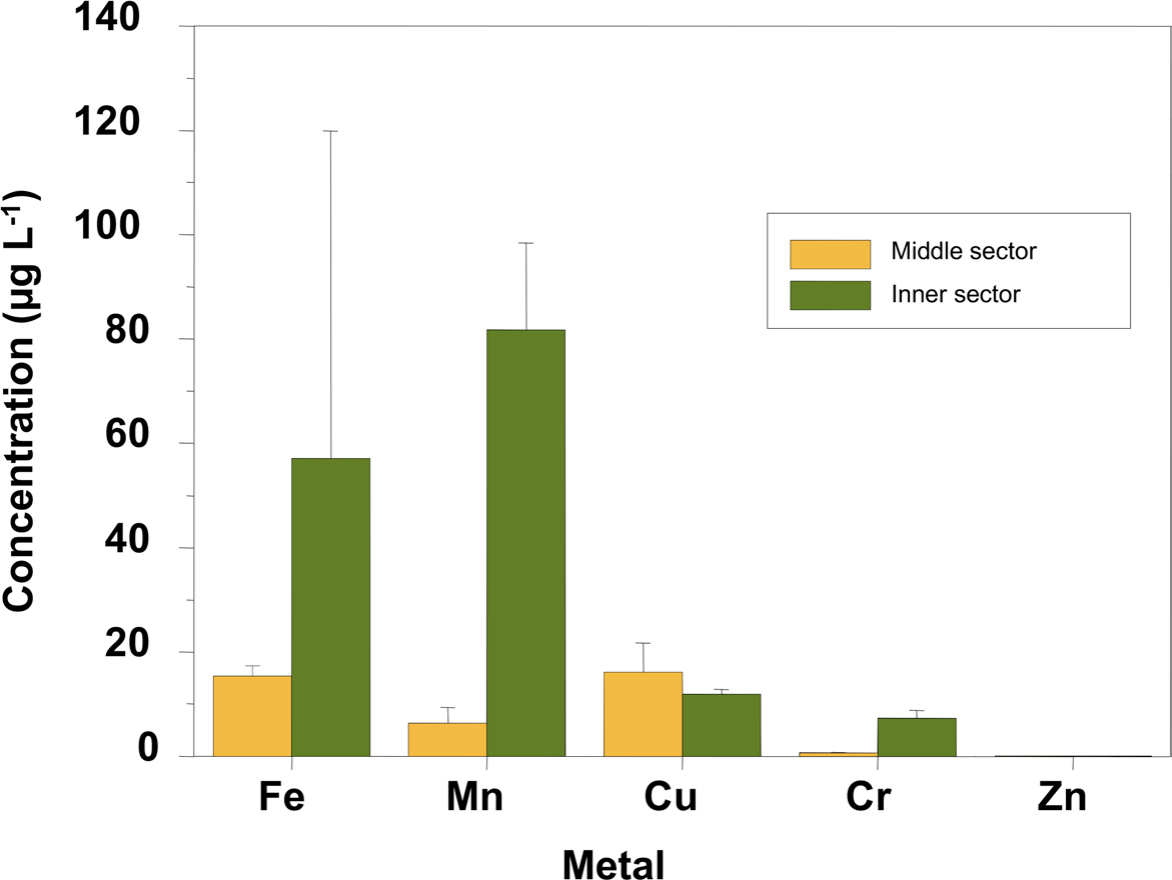
Concentration of trace elements released into the aqueous phase (mean values ± standard deviation) by sectors (P5 and P9, middle sector; P7 and PC, inner sector)

The concentration of trace elements in the aqueous phase varied depending on the element considered (Table 3). Hg and Pb were always below the detection limit during the experiment. On average, the sediments of the inner sector showed a higher net trace element release than those of the middle sector (Fig. 5).

**Table 3:** Trace metal concentrations in the aqueous phase during aerobic remobilization of sediments. Data reported in µg L^−1^

Sample	Time (days)	Fe	Mn	Cu	Cr	Zn
P5	0	bdl*	4.00 ± 0.07	6.75 ± 0.64	0.57 ± 0.06	0.011 ± 0.003
	1	5.25 ± 0.20	8.50 ± 0.71	25.30 ± 0.14	0.68 ± 0.04	0.011 ± 0.001
	5	2.00 ± 0.30	1.00 ± 0.04	11.65 ± 0.05	0.74 ± 0.07	0.011 ± 0.001
PC	0	bdl*	4.00 ± 0.07	6.75 ± 0.64	0.57 ± 0.06	0.011 ± 0.003
	1	101.50 ± 4.90	70.00 ± 2.83	11.50 ± 0.14	6.25 ± 0.21	0.030 ± 0.001
	5	bdl*	11.00 ± 0.71	12.74 ± 2.51	8.43 ± 0.15	0.045 ± 0.001
P7	0	bdl*	4.00 ± 0.07	6.75 ± 0.64	0.57 ± 0.06	0.011 ± 0.003
	1	13.25 ± 0.40	93.50 ± 4.95	10.91 ± 0.37	bdl*	0.013 ± 0.001
	5	bdl*	63.00 ± 4.24	12.58 ± 1.75	bdl*	0.015 ± 0.001
P9	0	bdl*	4.00 ± 0.07	6.75 ± 0.64	0.57 ± 0.06	0.011 ± 0.003
	1	7.90 ± 0.10	4.25 ± 0.35	10.13 ± 0.73	0.37 ± 0.02	0.011 ± 0.001
	5	bdl*	3.00 ± 0.09	11.65 ± 0.21	0.36 ± 0.09	0.011 ± 0.010

^*^bdl = below the detection limit

The release of Fe in sediments P5 and P9 (middle sector) was lower than in sediments P7 and PC (inner sector), with the highest amount of Fe released into the aqueous phase observed in the PC sample (Table 3). The dynamics of Fe release indicate that it was liberated very rapidly, reaching its maximum value at t1. Following this liberation of Fe, the concentrations decreased significantly until reaching undetectable levels in the aqueous phase (Table 3).

The Mn released into the aqueous phase followed a similar behavior to that observed for Fe. Sediments P7 and PC released the highest amounts of Mn into the aqueous phase (> 60 µg L^−1^), while samples P5 and P9 released a smaller amount of this trace element (< 8 µg L^−1^) (Table 3). The maximum values of Mn in the aqueous phase were determined on the first day of the experiment. Subsequent to this release, the concentrations of Mn in the aqueous phase decreased noticeably, though not as pronounced as in the case of Fe.

The release of Cu into the aqueous phase exhibited a different behavior compared to Fe and Mn. Cu release was continuous, with values increasing over time, except in the P5 sample, where the average value on day 1 (25.30 ± 0.14 µg L^−1^) was higher than on day 5 (11.65 ± 0.05 µg L^−1^) (Table 3).

The concentrations of Cr in the aqueous phase were very low. In samples P5 and PC, the dissolved Cr increased throughout the experiment. In the case of sample P5, it reached an average value of 0.74 ± 0.07 µg L^−1^ at t5, while in PC, it was 8.43 ± 0.15 µg L^−1^ (Table 3). On the other hand, Cr was not detected in the aqueous phase during the experiment in sample P7, while in sample P9, there was a decrease in concentration over time (Table 3).

Zinc exhibited very low concentrations in the original water sample, and the amount of this trace element transferred to the aqueous phase was also low. The values in the aqueous phase remained constant during the experiment in samples P5 and P9 (Table 3). In contrast, dissolved Zn was detected in samples P7 and PC with very low values (< 0.05 µg L^−1^) and a slight tendency to increase over time (Table 3).

### Geochemical fractions of trace elements in the sediments

The behavior of the total trace element concentrations during the experiment is shown in Table 4. The most relevant aspect is the decrease of the total trace element concentrations in all samples, being more pronounced on day 1. On day 5, the trace elements Fe, Cu, Zn, and Pb slightly increased their concentrations in some sediment samples, although they were still lower than the initial values, except for Fe in P5 and PC (Table 4). The remaining trace elements (Mn, Cr, and Hg) showed a decreasing trend over time in all samples. The percentages of trace elements in the reactive (F_react_), residual (F_sil_), organic (F_om_), and sulfide (F_pyr_) fractions, in relation to the values of the total concentration of trace elements in the sediment samples, are shown in Tables 5, 6, 7 and 8.

**Table 4:** Temporal variation of the total concentration of trace elements in the sediment during the experiment

Sample	Time (days)	Fe (mg g^−1^)	Mn (mg kg^−1^)	Cu (mg kg^−1^)	Cr (mg kg^−1^)	Zn (mg kg^−1^)	Hg (µg kg^−1^)	Pb (mg kg^−1^)
P5	0	16.04	94.03	86.33	36.34	86.32	88.51	26.48
	1	15.59	87.75	50.40	31.50	66.64	73.69	18.84
	5	18.07	87.67	58.34	31.48	79.53	69.70	18.41
PC	0	66.06	390.39	160.01	186.66	418.98	1755.08	239.88
	1	63.11	383.18	82.21	156.50	362.52	1285.74	175.34
	5	66.44	365.58	112.18	141.45	370.82	886.29	200.08
P7	0	20.17	142.34	9.88	64.91	70.90	63.49	24.57
	1	18.78	138.70	7.85	59.74	67.14	45.48	18.99
	5	18.43	131.82	5.42	51.76	64.38	17.31	22.09
P9	0	9.48	96.61	4.77	22.98	37.25	22.88	9.47
	1	9.05	90.52	3.59	21.29	33.94	15.47	7.53
	5	8.80	90.30	3.27	20.05	33.70	14.77	8.32

**Table 5:** Percentages of trace elements, relative to the total concentration, in the silicate-bound fraction (Fsil)

Metal	Time	Core P5	Core PC	Core P9	Core P7	Mean	Standard deviation
Fe	t0	46.26	60.88	68.59	54.99	57.68	9.43
	t1	47.00	62.61	70.30	58.94	59.71	9.71
	t5	40.91	59.22	72.66	60.15	58.23	13.07
Mn	t0	67.97	61.52	69.09	67.32	66.48	3.38
	t1	72.71	62.48	72.99	68.60	69.20	4.90
	t5	72.50	65.35	73.59	72.42	70.97	3.78
Cu	t0	4.15	10.04	0.00	0.00	3.55	4.75
	t1	6.99	18.92	0.00	0.00	6.48	8.93
	t5	6.04	13.71	0.00	0.00	4.94	6.51
Cr	t0	22.01	26.87	71.19	30.66	37.68	22.62
	t1	25.31	31.71	74.59	32.91	41.13	22.56
	t5	25.32	34.73	79.14	38.43	44.41	23.80
Zn	t0	21.87	35.87	74.81	64.95	49.37	24.68
	t1	28.13	41.41	81.41	67.07	54.51	24.14
	t5	23.67	40.25	82.54	69.95	54.10	26.94
Hg	t0	0.00	0.00	0.00	0.00	0.00	0.00
	t1	0.00	0.00	0.00	0.00	0.00	0.00
	t5	0.00	0.00	0.00	0.00	0.00	0.00
Pb	t0	6.44	3.09	16.61	5.81	7.99	5.93
	t1	8.75	4.23	20.78	7.47	10.30	7.24
	t5	9.00	3.70	18.65	6.38	9.43	6.52

**Table 6:** Percentages of trace elements, relative to the total concentration, in the reactive fraction (F_react_)

Metal	Time	Core P5	Core PC	Core P9	Core P7	Mean	Standard deviation
Fe	t0	45.95	20.38	0.04	12.09	19.61	19.44
	t1	48.70	21.62	0.04	13.12	20.87	20.57
	t5	55.53	27.41	0.06	14.47	24.37	23.59
Mn	t0	17.63	16.08	5.43	8.49	11.91	5.88
	t1	19.22	15.69	6.36	7.93	12.30	6.16
	t5	20.90	16.33	6.76	8.70	13.17	6.60
Cu	t0	81.92	61.30	2.55	8.34	38.53	39.19
	t1	75.90	50.06	1.16	3.16	32.57	36.67
	t5	87.01	64.52	1.92	12.10	41.39	40.96
Cr	t0	34.31	7.49	0.50	3.30	11.40	15.54
	t1	42.29	10.68	0.56	3.93	14.36	19.09
	t5	50.70	12.59	0.66	5.13	17.27	22.82
Zn	t0	75.10	52.23	1.83	14.25	35.85	33.83
	t1	70.39	45.61	0.67	13.68	32.59	31.49
	t5	73.60	50.11	1.29	13.25	34.56	33.30
Hg	t0	48.22	0.08	0.59	0.39	12.32	23.93
	t1	57.63	0.15	0.77	0.48	14.76	28.59
	t5	66.95	0.24	1.39	2.42	17.75	32.81
Pb	t0	70.86	77.78	15.37	39.38	50.85	28.96
	t1	67.34	74.15	7.87	32.02	45.35	31.07
	t5	75.50	77.07	13.06	40.37	51.50	30.72

**Table 7:** Percentages of trace elements, relative to the total concentration, in the reactive organic fraction (F_mo_)

Metal	Time	Core P5	Core PC	Core P9	Core P7	Mean	Standard deviation
Fe	t0	5.35	8.86	22.06	14.70	12.74	7.31
	t1	2.64	8.05	21.64	14.58	11.73	8.22
	t5	2.14	7.39	20.79	13.13	10.86	8.00
Mn	t0	13.97	21.99	22.74	21.00	19.92	4.03
	t1	7.71	21.50	18.45	20.43	17.02	6.33
	t5	6.35	17.99	17.45	15.96	14.44	5.46
Cu	t0	10.80	17.65	79.49	21.39	32.33	31.74
	t1	15.17	15.85	76.57	23.73	32.83	29.42
	t5	5.57	11.14	79.92	18.36	28.75	34.51
Cr	t0	43.28	63.66	27.19	63.42	49.39	17.61
	t1	32.20	55.62	23.76	60.49	43.02	17.81
	t5	23.85	50.95	19.24	53.94	37.00	17.98
Zn	t0	2.78	10.46	22.03	17.03	13.07	8.34
	t1	1.28	11.64	16.94	15.96	11.46	7.16
	t5	2.65	8.62	15.28	14.33	10.22	5.84
Hg	t0	16.25	1.71	4.72	0.00	5.67	7.32
	t1	10.06	0.60	5.88	0.00	4.14	4.75
	t5	11.83	2.70	1.95	0.00	4.12	5.26
Pb	t0	15.66	15.15	62.49	44.78	34.52	23.23
	t1	20.53	17.90	66.54	50.91	38.97	23.71
	t5	14.40	17.36	63.96	46.05	35.44	23.77

**Table 8:** Percentages of trace elements, relative to the total concentration, in the pyritic fraction (Fpyr)

Metal	Time	Core P5	Core PC	Core P9	Core P7	Mean	Standard deviation
Fe	t0	2.45	9.87	9.32	18.22	9.97	6.45
	t1	1.66	7.72	8.02	13.36	7.69	4.78
	t5	1.37	5.97	6.48	12.25	6.52	4.46
Mn	t0	0.42	0.42	2.75	3.18	1.69	1.48
	t1	0.35	0.33	2.21	3.04	1.48	1.36
	t5	0.25	0.32	2.20	2.92	1.42	1.35
Cu	t0	3.13	11.01	17.96	70.27	25.59	30.39
	t1	1.94	15.16	22.27	73.11	28.12	31.15
	t5	1.38	10.63	18.16	69.55	24.93	30.53
Cr	t0	0.40	1.98	1.12	2.62	1.53	0.98
	t1	0.20	1.99	1.08	2.67	1.49	1.08
	t5	0.13	1.73	0.96	2.50	1.33	1.02
Zn	t0	0.25	1.44	1.33	3.77	1.70	1.48
	t1	0.20	1.33	0.98	3.29	1.45	1.31
	t5	0.08	1.01	0.89	2.47	1.11	1.00
Hg	t0	35.53	98.21	94.70	99.61	82.01	31.06
	t1	32.30	99.25	93.35	99.52	81.11	32.66
	t5	21.22	97.06	96.65	97.58	78.13	37.94
Pb	t0	7.03	3.97	5.53	10.03	6.64	2.58
	t1	3.38	3.73	4.80	9.60	5.38	2.88
	t5	1.10	1.88	4.33	7.20	3.63	2.75

**Fig. 6:**
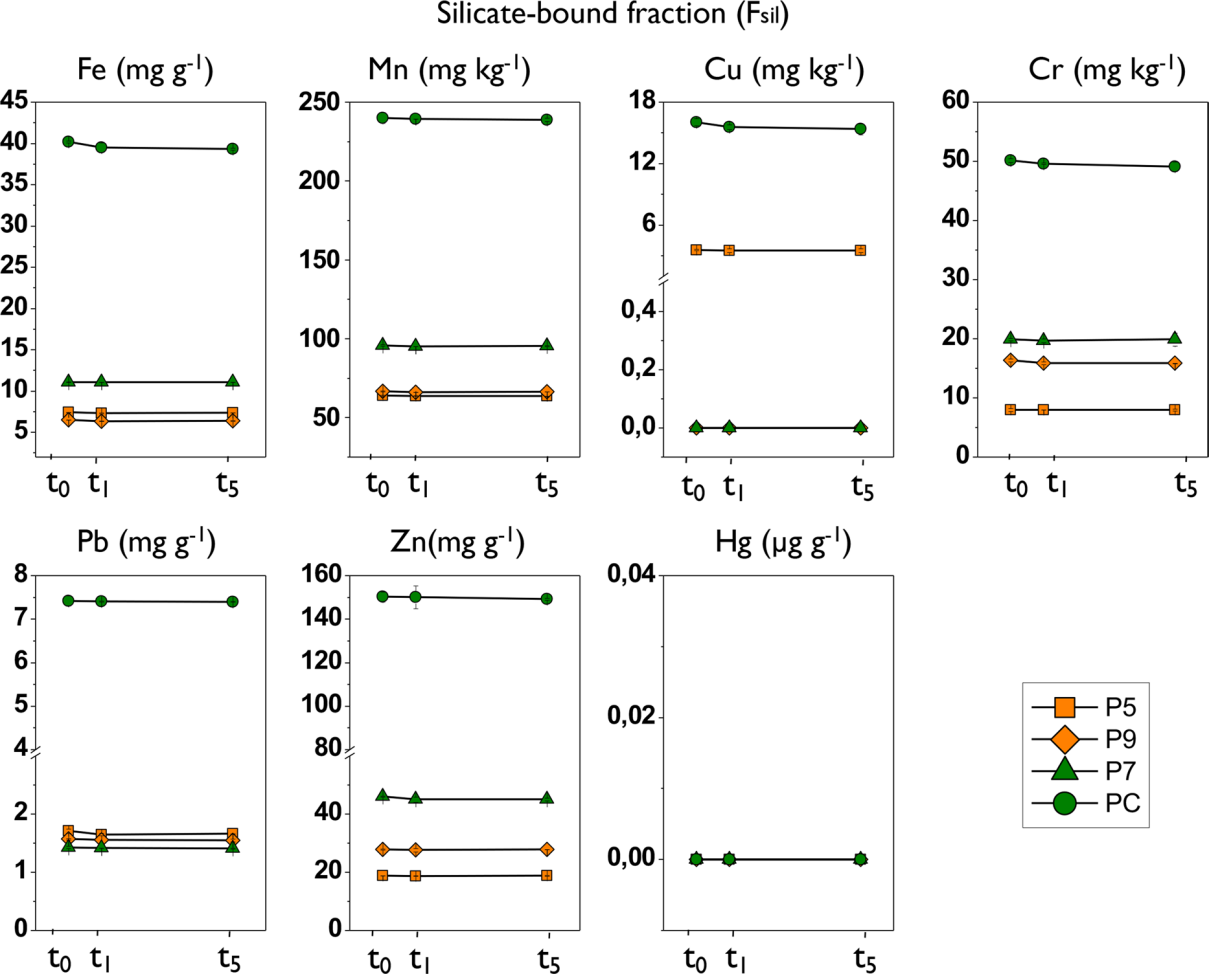
Results of the temporal variation in the concentration of trace elements in the silicate-bound fraction (F_sil_) in the sediment

When considering the different geochemical fractions of the sediment, it is observed that the concentrations of trace elements in the F_sil_ remained practically constant throughout the experiment: Fe, Mn, Cr, and Zn presented the highest average percentages of this fraction with respect to the total (> 36%), while Cu and Pb registered very low percentages (< 10%) and finally Hg was not detected in this fraction (Fig. 6; Table 5).

**Fig. 7:**
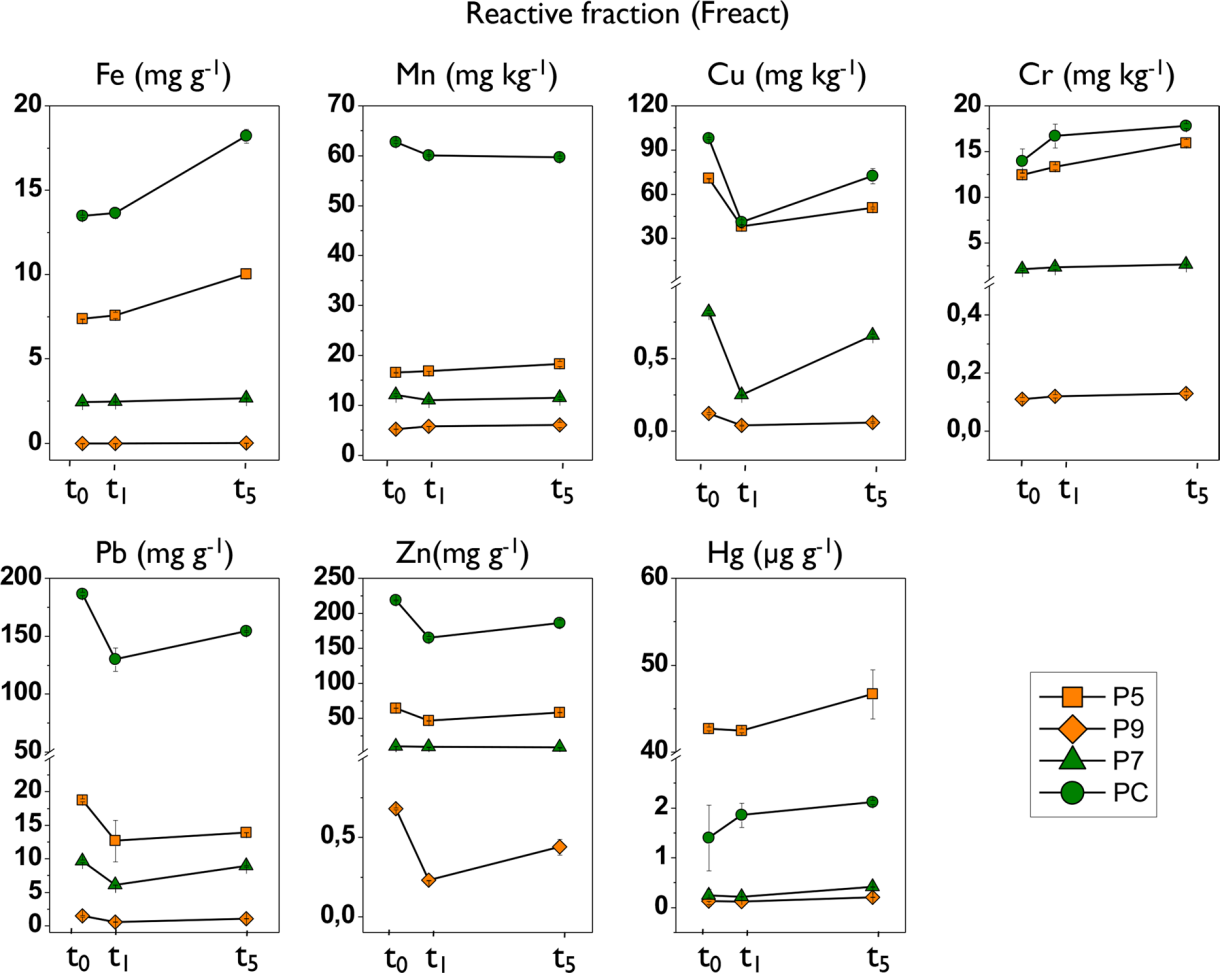
Results of the temporal variation in the concentration of trace elements in the reactive fraction (F_react_) in the sediment

For F_react_, Cu, Zn, and Pb presented the highest mean percentages (> 35%) in the sediment sample at t0, with the highest mean value for Pb (> 50%). The rest of the trace elements were below 20% in the samples (Fig. 7; Table 6). The time variation of trace elements in this fraction progressively increased for Fe, Mn, Cr, and Hg, while Cu, Zn, and Pb decreased at t1. At t5, the content of the latter trace elements increased in this fraction of the sediment, but with slightly lower values than those recorded prior to oxidation (t0).

**Fig. 8:**
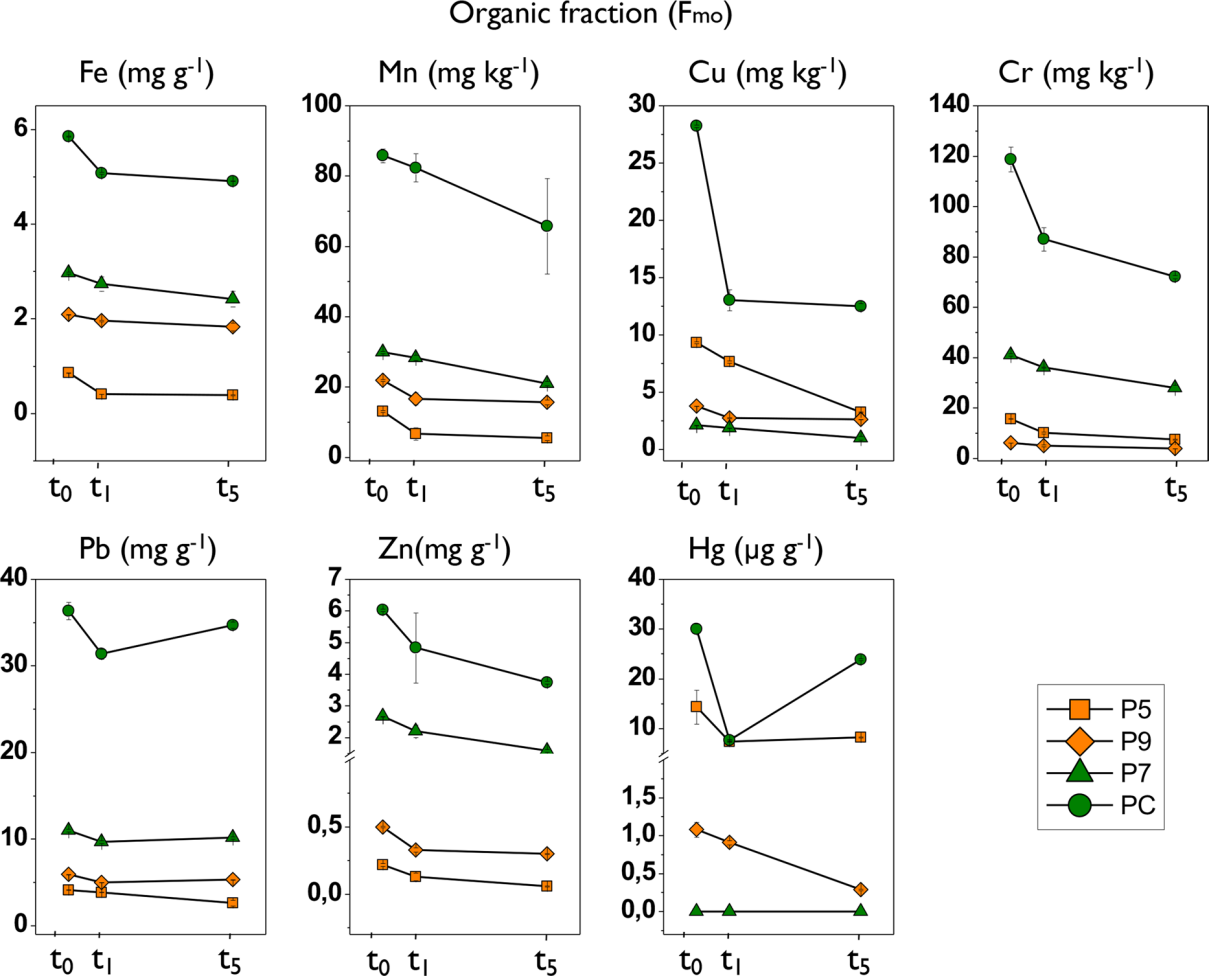
Results of the temporal variation in the concentration of trace elements in the organic fraction (F_mo_) in the sediment

For F_mo_, the average proportion of Cu, Cr, and Pb at time zero was 30%, while for the rest of the trace elements it was less than 20% (Fig. 8; Table 7). At t1, the concentration of all trace elements in this fraction decreased, in some cases to more than 50% of their initial value. At t5, a slight increase was observed in this fraction in the concentrations of most trace elements, but their values were still lower than the initial values (Fig. 8; Table 7).

**Fig. 9:**
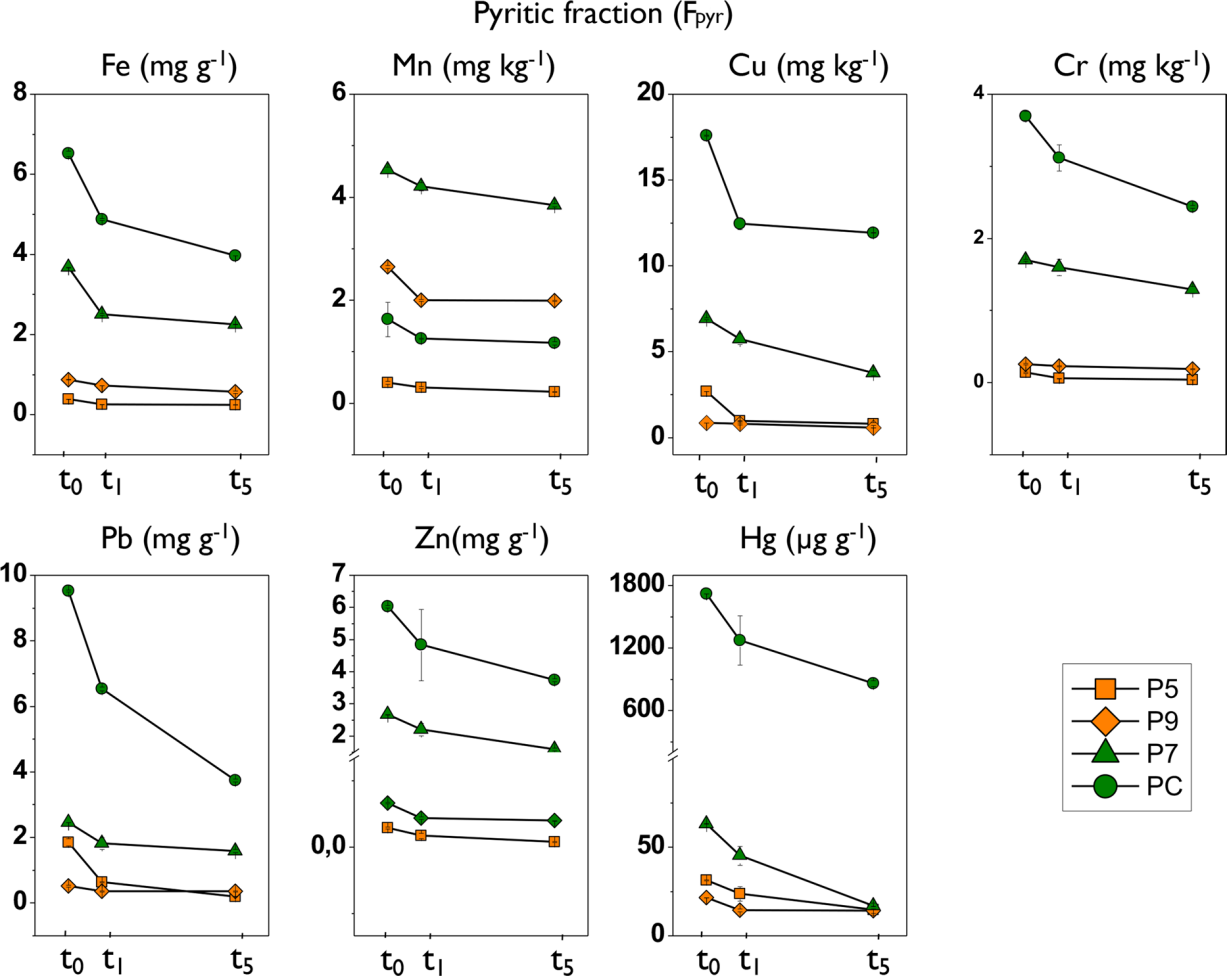
Results of the temporal variation in the concentration of trace elements in the pyritic fraction (F_pyr_) in the sediment

The concentration of trace elements in the F_pyr_ initially (t0) was very variable between elements. Mn, Cr, and Zn had the lowest average percentages (< 2%). While Cu and Hg had the highest concentrations in this fraction (> 25%) (Fig. 9; Table 8). With intermediate values, around 10%, Fe and Pb stand out. It should be noted that practically all the Hg was found in the form of sulfide (> 80% average value). Regarding the temporal evolution of the trace elements associated with this fraction, a continuous and progressive decrease in trace element concentration was observed over time for all samples, indicating that this fraction of the sediment is the most susceptible to aerobic oxidation.

## Discussion

### pH variation in the aqueous phase

The decrease in pH in an oxygen-saturated aqueous environment is one of the consequences of the presence of oxidizable compounds in the sediment, which generate acidity, as suggested by Rimstidt and Vaughan (2003). These oxidizable substances in the sediment include sedimentary sulfides and organic matter.

The magnitude of pH variations in seawater in contact with sediment samples (Fig. 4), which is most pronounced in the inner sediments and less significant in the middle sector, is closely related to the amount of metallic sulfides, organic matter, and carbonates present in the sediments of the Ría de Pontevedra. Sediment samples from the inner sector exhibit more anoxic conditions with a predominance of pyrite and other mineral sulfides with pyritization levels exceeding 60% (León et al., 2004), in contrast to the middle sector, which is oxic-suboxic in nature, with values around 30%. Besides the lower pyrite concentration, the higher carbonate concentration in the middle sector of the estuary may contribute to this smaller pH decrease since carbonates act as efficient buffers at these pH levels. The slight pH increase on the fifth day might initially suggest a decrease in the oxidation process despite the continued vigorous airflow. However, sediment oxidation continued, as reflected in the decreasing values of trace elements associated with sulfides and organic matter in the sediments. Therefore, the slight pH increase on the fifth day is attributed to the strong buffering capacity of seawater and potential dissolution of carbonates.

### Behavior of trace element oxidation

The decrease in the total trace element concentration in the sediment is another piece of evidence, along with the pH decrease, of the presence of oxidizable phases in the sediments of the Ría de Pontevedra. The lowest total concentrations of heavy trace elements were recorded in the middle sector sediments, specifically in sample P9. In contrast, the highest concentrations were found in the inner sector of the estuary, primarily in sample PC (Table 4). The inner sectors of the Rías Baixas are characterized by estuarine conditions, whereas the middle and outer sectors can be considered purely marine (Vilas et al., 2019). Furthermore, the inner sector is more influenced by anthropogenic inputs and has a higher content of silt and organic matter than other parts of the estuary, two factors that contribute to increasing the trace element concentration in the sediment (Rubio et al., 2001).

The rapid release of all trace elements from the sediment into the aqueous phase at the beginning of the experiment (t1) was facilitated by the decrease in pH. In contrast, at t5, the trace elements exhibited a withdrawal from the solution, which coincided with an increase in pH. The removal of trace elements from the aqueous phase, especially for Mn and Fe, is attributed to the rapid formation of Fe and Mn oxides and oxyhydroxides that precipitate or form coatings on the sediments.

Initially, the trace elements present in the F_mo_ and F_pyr_ were primarily oxidized and released into the aqueous phase. On the other hand, the trace elements in the F_sil_ did not exhibit significant changes in their concentrations throughout the experiment, suggesting that the oxidation of trace elements in this fraction can be considered negligible. Trace elements in the F_react_ of the sediment may have transferred to the aqueous phase during the early stages of the experiment, but it is highly likely that they precipitated by the end of the experiment, associated with the newly formed oxidized compounds of Fe and Mn.

Fe and Mn are the trace elements that control the temporal behavior of the other trace elements present in the aqueous phase. Fe and Mn, in their reduced forms, are Fe (II) and Mn (II), and these chemical forms are stable only under acidic conditions. The decrease in pH at t1 likely favored the release of Fe and Mn into the aqueous phase, along with the release of other trace elements. As the oxygen pumping system continued, the Fe^2+^ and Mn^2+^ ions oxidized to form Fe(III) and Mn(III, IV).

Therefore, the formation of Fe and Mn oxides and oxyhydroxides generally regulates the presence of certain trace elements like Hg and Pb, preventing their release into the aqueous phase. However, they did not act as sinks for other trace elements such as Zn, Cr, and especially Cu, whose concentrations during the experiment increased considerably. The maximum concentration of total dissolved Cu is higher than that indicated by other authors as a toxicity threshold (> 8.9 µg L^−1^) (Beiras & Albentosa, 2004) for organisms in the Galician estuaries. These results indicate that the aerobic oxidation of the analyzed sediments is an important process that elevates the concentrations of this trace element in the aqueous phase. The increase in the transfer of Cu to seawater in oxygenated sediments has also been observed in previous studies in the Ría of Vigo (Santos-Echeandia et al., 2009). This highlights the potential danger posed by sediment resuspension in the study area. It is important to consider that Galicia is the world’s leading producer and marketer of mussels and second in terms of production and extraction of this bivalve. For this reason, the regional administration approves triennial shellfish and fishing exploitation plans (General Shellfish Exploitation Plan for the 2024–2026 triennium). These plans regulate management strategies to ensure sustainable exploitation of shellfish resources. The significance and validity of our work lies in the fact that these management plans included in the General Plan encompass biological, ecological, economic, and social objectives, with reference levels and indicators, as well as action strategies. Among these strategies, the role of dredging, along with sediment remobilization and oxidation, is a key factor to be considered, as already suggested by other studies conducted in these estuarine environments (Norén et al., 2020; Rodríguez-Romero et al., 2016).

## Conclusions

The aerobic oxidation of sediments in the Ría de Pontevedra led to the release of trace elements into the marine aqueous phase, as evidenced by the decrease in the total concentration of trace elements and the oxidizable sediment fractions (F_mo_ and F_pyr_). Trace element release into the aqueous phase was the predominant mechanism on day 1, while their removal was the dominant process on day 5. The oxidized chemical species of Fe and Mn not only self-regulate their concentrations in the aqueous environment but also control the presence of other trace elements in it. Cu was the only trace element that was released in quantities sufficient to be toxic to marine organisms. It can be incorporated into filter-feeding and detritivore organisms, of commercial interest by reaching concentrations capable of producing biological effects and bioaccumulating in animal tissues.

Galicia leads globally in mussel production and ranks second in bivalve extraction. Triennial shellfish and fishing exploitation plans (General Shellfish Exploitation Plan for 2024–2026) are crucial for the management of the Galician Rías. These plans, covering biological, ecological, economic, and social aspects, ensure sustainable resource management through defined objectives, indicators, and action strategies. For this reason, the type of control carried out in this research should be considered in environmental impact studies and should be transferred to stakeholders when carrying out any anthropogenic activity that may affect the cultivation and wealth of shellfish in the ría environments.

## Data Availability

No datasets were generated or analysed during the current study.
